# Estimating the Impact of Workplace Bullying: Humanistic and Economic Burden among Workers with Chronic Medical Conditions

**DOI:** 10.1155/2015/708908

**Published:** 2015-10-18

**Authors:** A. Fattori, L. Neri, E. Aguglia, A. Bellomo, A. Bisogno, D. Camerino, B. Carpiniello, A. Cassin, G. Costa, P. De Fazio, G. Di Sciascio, G. Favaretto, C. Fraticelli, R. Giannelli, S. Leone, T. Maniscalco, C. Marchesi, M. Mauri, C. Mencacci, G. Polselli, R. Quartesan, F. Risso, A. Sciaretta, M. Vaggi, S. Vender, U. Viora

**Affiliations:** ^1^Department of Clinical Sciences and Community Health, University of Milan, 20124 Milan, Italy; ^2^Department of Psychiatry, University of Catania, 95131 Catania, Italy; ^3^Department of Clinical and Experimental Medicine, University of Foggia, 71121 Foggia, Italy; ^4^Department of Mental Health, UO Cava de'Tirreni, 84013 Salerno, Italy; ^5^Department of Public Health, Clinical and Molecular Medicine, University of Cagliari, 09124 Cagliari, Italy; ^6^Department of Mental Health, ASS n.6, 33170 Pordenone, Italy; ^7^IRCCS Maggiore Policlinico Hospital, Ca'Granda Foundation, 20124 Milan, Italy; ^8^Department of Health Sciences, School of Specialization in Psychiatry, Magna Græcia University of Catanzaro, 88100 Catanzaro, Italy; ^9^Psychiatric Neuroscience Group, Department of Neurological and Psychiatric Sciences, University of Bari, 70125 Bari, Italy; ^10^Department of Mental Health, ULSS 7, Conegliano, 31015 Treviso, Italy; ^11^Unit of Psychiatry, Sant'Anna Hospital, 22100 Como, Italy; ^12^National Association of Rheumatic Patients (ANMAR), 00153 Rome, Italy; ^13^National Association for Inflammatory Bowel Disease (AMICI), 20131 Milan, Italy; ^14^Department of Mental Health, AULSS Legnago Hospital, Legnago, 37045 Verona, Italy; ^15^Department of Neuroscience, Psychiatry Unit, University of Parma, 43121 Parma, Italy; ^16^Mental Health Department, AUSL, 43126 Parma, Italy; ^17^Department of Experimental and Clinic Medicine, Section of Psychiatry, University of Pisa, 56100 Pisa, Italy; ^18^Depression Unit, Neuroscience Department, Fatebenefratelli Hospital, 20137 Milan, Italy; ^19^Department of Neurology and Psychiatry, Policlinico Umberto I, Sapienza University of Rome, 00185 Rome, Italy; ^20^Division of Psychiatry, Clinical Psychology and Psychiatric Rehabilitation, Department of Medicine, New Faculty of Medicine, University of Perugia, Sant'Andrea delle Fratte, 06156 Perugia, Italy; ^21^Unit of Psychiatry, A.S.L. CN1, 12100 Cuneo, Italy; ^22^Servizio Psichiatrico di Diagnosi e Cura (SPDC), Tivoli Hospital, 00019 Rome, Italy; ^23^Department of Mental Health, ASL3, 16125 Genoa, Italy; ^24^Department of Clinical and Experimental Medicine, Psychiatry, Faculty of Medicine and Surgery, University of Insubria, 21100 Varese, Italy; ^25^National Association ANAP Onlus, Rivoli, 10098 Turin, Italy

## Abstract

*Background*. Although the prevalence of work-limiting diseases is increasing, the interplay between occupational exposures and chronic medical conditions remains largely uncharacterized. Research has shown the detrimental effects of workplace bullying but very little is known about the humanistic and productivity cost in victims with chronic illnesses. We sought to assess work productivity losses and health disutility associated with bullying among subjects with chronic medical conditions. *Methods*. Participants (*N* = 1717) with chronic diseases answered a self-administered survey including sociodemographic and clinical data, workplace bullying experience, the SF-12 questionnaire, and the Work Productivity Activity Impairment questionnaire. *Results*. The prevalence of significant impairment was higher among victims of workplace bullying as compared to nonvictims (SF-12 PCS: 55.5% versus 67.9%, *p* < 0.01; SF-12 MCS: 59.4% versus 74.3%, *p* < 0.01). The adjusted marginal overall productivity cost of workplace bullying ranged from 13.9% to 17.4%, corresponding to Italian Purchase Power Parity (PPP) 2010 US$ 4182–5236 yearly. Association estimates were independent and not moderated by concurrent medical conditions. *Conclusions*. Our findings demonstrate that the burden on workers' quality of life and productivity associated with workplace bullying is substantial. This study provides key data to inform policy-making and prioritize occupational health interventions.

## 1. Introduction 

All developed countries are facing a sustained shift in the demographic composition of their population and are thus devoting major effort to increasing the work participation rate of aging and disabled people [1]. Together with long-term health problems and chronic diseases, prevalence of work-limiting disabilities increases with age [2]: it has been estimated that 72% of all-causes Disability-Adjusted Life Years occur in subjects under 60 years old and more than three-quarters of old workers have at least one chronic health condition that requires management [3, 4]. In addition, the majority of workers with chronic illnesses continue to work and have to deal with several workplace risk factors [5, 6]. However, the interplay between occupational exposures and chronic medical conditions remains largely uncharacterized, thus limiting the potential for effective preventive and therapeutic actions.

Workplace bullying is a common and severe occupational stressor and imbalance of power, harm, and systematic repetition over time represent its key elements [7]. The adverse effects of workplace bullying on victims' psychological health span from mild anxiety and depression to severe posttraumatic stress symptoms [8–14]. Similarly, workplace bullying also has a detrimental impact on organizational outcomes, such as job satisfaction, organizational commitment, and intention to leave [15, 16].

Despite evidence showing that workplace bullying may be associated with a significant financial burden for victims and organizations, cost estimates are difficult to compare due to different currencies, methodologies, time frames, and the selection of different cost drivers (e.g., health care cost, productivity and performance loss, sick leave, and replacement costs) [17].

Research has shown that having a disability is a risk factor for being bullied [18–21] and there is some evidence showing longitudinal associations between mental health problems and subsequent exposure to bullying at work [22–25]. Targets of workplace bullying with preexisting chronic diseases could experience worse consequences and perceive themselves as being bullied more frequently compared to colleagues with no other medical conditions [12, 26]. Furthermore, exposure to psychosocial stressors at work may play an important role in retirement behavior and labor supply decisions among workers with chronic diseases [27, 28].

Although psychosocial factors and chronic conditions are both emerging issues in occupational medicine, very little is known about the humanistic and productivity cost of bullying at work in workers with chronic illness. Empirical research would help health authorities and employers prioritize the allocation of limited resources for occupational health interventions [29]. As a consequence, the assessment of health-related quality of life and cost-effectiveness analyses are gaining importance in occupational medicine because decision-makers need comparable and accurate information in order to achieve the greatest health improvement for their workforce. In the present study, we sought to evaluate work productivity losses and health disutility associated with workplace bullying among patients with different chronic medical conditions.

## 2. Materials and Methods

### 2.1. Participants and Procedures

The present study is a joint analysis of the Liberamente and MOSAICO research datasets. Both studies aimed at evaluating the quality of life, treatment satisfaction, social participation, and health care utilization of patients with common medical conditions such as major depression disorder (Liberamente study), inflammatory bowel disease, psoriasis and autoimmune arthritis (MOSAICO study).

The Liberamente study was carried out between June and July 2013 in 18 outpatient referral centers for diagnosis and treatment of psychiatric disorders across all Italian regions. Patients referred to the centers for psychiatric conditions were screened for eligibility by a psychiatrist during a regular follow-up visit at the clinic. We included adult patients with a clinical diagnosis of depression (i.e., recurrent depressive disorder, major depressive episode, adjustment disorder, mixed affective disorder, dysthymia, and other persistent depressive disorders) with the exclusion of bipolar disorders. Seven hundred patients agreed to participate in the research completing an anonymous self-administered paper-and-pencil questionnaire. Concurrently, the same psychiatrist recorded relevant clinical characteristics in a standardized data collection form. To preserve anonymity of data collection while matching clinical and patient-reported information, the psychiatrist handed the data collection form to the patient at the end of the visit. The patients sealed both the data collection form and the self-administered questionnaire in an anonymous envelope to return to the research team.

The MOSAICO study was carried out between April and October 2014. We invited the members of Patients' Associations for people with Crohn's Disease, ulcerative colitis, psoriasis, ankylosing spondylitis, rheumatoid arthritis, and psoriatic arthritis to take part in the study. The survey adopted a dual methodology. A web survey was posted on the associations' web sites. Respondents were 143 registered patients with autoimmune arthritis, 397 patients with inflammatory bowel disease, and 236 patients with psoriasis. All participants accessed the survey from their personal account. Additionally, 152, 52, and 37 patients with autoimmune arthritis, inflammatory bowel disease, and psoriasis, respectively, elected to complete a self-administered paper-and-pencil version of the survey.

### 2.2. Measures

Surveys included sociodemographic and occupational information, the Work Productivity and Activity Impairment scale, and the SF-12v1 (RAND).

#### 2.2.1. Workplace Bullying

Research has essentially used two methodologies to assess workplace bullying: (i) investigating subjects' perceptions of victimization (self-labelling method) and/or (ii) their exposure to specific bullying behaviors (behavioral experience method) [30]. In this study, workplace bullying was measured using the self-labelling method by providing respondents with a theoretical definition of workplace bullying (“bullying takes place when one or more persons systematically and over time feel that they have been subjected to negative treatment on the part of one or more persons, in a situation in which the person(s) exposed to the treatment have difficulty in defending themselves against them. It is not bullying when two equally strong opponents are in conflict with each other” [31, pages 190-191]). Participants responded to a single-item question (“according to this definition have you been subjected to bullying at the workplace during the last six months?”) using a five-point scale from 1 (never) to 5 (yes, many times a week). Subjects reporting a frequency of bullying of 3 or above on the five-point scale were considered victims of workplace bullying for the purpose of this analysis. In addition, the length of time for which workplace bullying had been experienced was assessed.

#### 2.2.2. Outcomes

*Health-Related Quality of Life*. The SF-12 questionnaire (RAND, [32]) is a 12-item generic health profile measure assessing patients' perception of their own mental and physical health. Ratings use a 0–100-point scale. A *t*-score calculated on the normative values of the Italian general population is obtained from raw scores. Patients reporting scores lower than 42.0 and 43.85 on the SF-12 mental and physical composites, respectively, were classified as significantly impaired [33, 34].

*Health Utility*. The SD-6D utility index represents the value assigned to a specific health status characterized by the impairments, functional states, perceptions, and social opportunities that are influenced by disease, injury, treatment, or policy. The scoring algorithm of the Short Form 6 Dimension (SF-6D) is a two-step process: in the first step, responses to SF-12 questions are used to define a response vector representing the patient's health state (classification system); then, in the second step, the vector is converted into a utility value using a utility function obtained from a sample of the general population. The SF-6D classification system includes six multilevel dimensions (physical functioning, role of participation, social functioning, bodily pain, mental health, and vitality) and describes 18,000 health states [35]. The SF-6D utility index was calculated according to the utility function observed by Brazier et al. [35] using standard gamble experiments carried out in a sample of the general population. Scale ratings range from 0 (death) to 1 (perfect health).

*Productivity Loss*. Economic evaluations conducted from an employer's perspective express the benefit of occupational health interventions in terms of health-related productivity, which is translated into a monetary value and may also be referred to as an indirect cost [29]. The Work Productivity Activity Impairment questionnaire [36] consists of 4 items: (Q1) hours lost due to health problems; (Q2) hours lost due to any other reason; (Q3) hours actually worked; and (Q4) the degree of which health problems affected productivity while at work. Responses ranged from 0 (“My health problem had no effect on my work”) to 10 (“My health problem completely prevented me from working”). Estimation metrics were calculated as percentage productivity losses, with higher values indicating a greater proportion of time lost at work (less productivity). The following equations were calculated.

Equation for sick leave is as follows:

(1) $$\begin{array}{c} \left[\;\frac{Q1}{\left(Q1+Q3\right)}\;\right]*100. \end{array}$$

Equation for work impairment while at work or presenteeism is as follows:

(2) $$\begin{array}{c} Q4*10. \end{array}$$

Equation for overall work productivity loss is as follows:

(3) SickLeave+1−SickLeave∗Presenteeism∗100.

Percentage productivity losses were converted to the corresponding share of the Italian Purchase Power Parity (PPP) per capita Gross Domestic Product (2010, US$ 31,090) [37] which allows cross-national comparisons. PPP represents the real exchange rate (nominal exchange rate adjusted for the price index), that is, how much money would be needed to purchase the same goods and services in two different countries.

#### 2.2.3. Demographic Information

Surveys included a section on sociodemographic characteristics. We recorded patients' age, gender, education level, marital status, employment status, employment contract, and preeminent job demand (either physical, mental, or mixed) with items from the Work Ability Index (WAI, [38]). Workforce status was defined based on patients' age (between 18 and 65 years). We classified employment, inactivity, retirement, and unemployment status using the International Labour Office definition [39]. Common medical information in both datasets included the number of days of hospitalization in the past 12 months, time since chronic disease onset (years), and chronic condition type (major depression disorder (MDD), autoimmune arthritis (AA), psoriasis (PSO), and inflammatory bowel disease (IBD)). We used number of days of hospitalization rather than overall health care utilization rates (i.e., outpatients visits, mental health services) as a proxy of chronic disease severity to minimize the information bias due to the inability to discern between bullying-related medical encounters and those caused by the cooccurring chronic medical condition.

### 2.3. Analysis

We computed means and standard deviation or absolute and relative frequency of continuous and categorical variables, respectively. We evaluated differences in sociodemographic and clinical characteristics across diagnosis status with *χ* ^2^ or one-way ANOVA as appropriate. Unadjusted and adjusted quality-of-life penalty, health disutility, and productivity losses associated with workplace bullying were estimated with generalized linear models. To account for the skewed distribution of outcomes, we fitted OLS regressions with log link function in the analysis of SF-12 and SF-6D scores. Additionally, we fitted gamma regressions for the analysis of lost productivity time (WPAI metrics). All models were adjusted for patients' age, gender, education, marital status, job demand, contract, hospitalization days, diagnosis, time since disease onset, and time since the onset of workplace bullying. We also tested the interaction between chronic disease and self-reported bullying experience in all models. *p* < 0.05 was considered statistically significant. Analyses were conducted with SAS 9.2.

## 3. Results

### 3.1. Sample Characteristics

Demographic and clinical characteristics of the sample are summarized in Table 1. Although the majority of subjects were of working age (46.8 ± 13.1), only 56% of the sample were actually employed. Participants with a paid job were more likely to be men (63.7% versus 51.3%, *p* < 0.01), were slightly younger (44.0 ± 9.8 versus 46.3 ± 12.5, *p* < 0.01), reported less hospitalization days (1.8 ± 6.5 versus 3.2 ± 10.4, *p* < 0.01), and were more likely to have tertiary qualifications (24.7% versus 13.6%, *p* < 0.01). Among subgroups, significant differences were seen in employment status: subjects with MDD and PSO reported, respectively, the highest percentage of inactivity (25.8%) and unemployment (17.4%), while 28.4% of patients with AA were retired. In most subgroups, there was greater representation of women and the overall prevalence of jobs with considerable physical demands was quite low. Apart from MDD, with most subjects reporting a more recent diagnosis (5.89 ± 7.38), most other chronic diseases had been affecting participants for a long time (12.4 to 13.3 years).

### 3.2. Prevalence of Workplace Bullying

Table 2 shows the prevalence of workplace bullying in the whole sample. One hundred and twenty-three subjects (16.3%) labelled themselves as victims of bullying at work. Bullied subjects were slightly older (44.6 ± 10.9 versus 47.2 ± 12.3; *p* = 0.02). No statistically significant differences were found in bullying prevalence across the different chronic diseases (AA 16.2%, IBD 15.4%, MDD 17.6%, and PSO 15.1%, *p* = 0.89).

Eighty-one percent of bullied subjects had a preexistent medical condition before bullying onset. However, in the subgroup of patients with MDD, 30% reported that workplace bullying had occurred before the onset of depression.

### 3.3. Workplace Bullying and Productivity Losses

**Figure 1:**
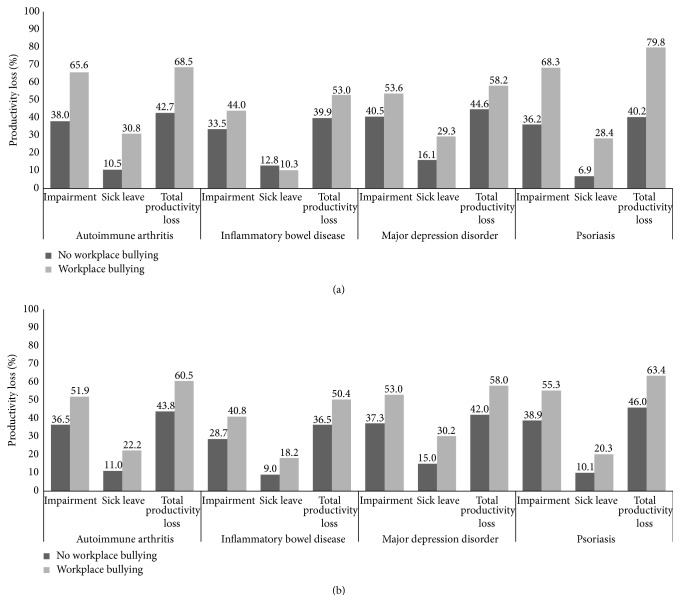
Work productivity losses across workplace bullying status among workers with different chronic conditions. (a) Unadjusted scores. (b) Adjusted scores. Models included age, gender, time since diagnosis, time since workplace bullying onset, education, marital status, job demand, contract, diagnosis, and hospitalization days.

The mean average weekly full-time equivalent sick hours were 6.58 ± 11.92, and corresponding average sick-leave rate was 16.4%  ±  29.8. Work impairment was 41.9%  ±  31.6, whereas the overall productivity loss (absenteeism + presenteeism) was 46.5%  ± 33.2. Unadjusted productivity losses due to sick leave and presenteeism were both associated with workplace bullying (Figure 1(a), *p* < 0.001). These associations were both robust to adjustment for possible confounders (Figure 1(b), *p* < 0.001) and were not moderated by disease status (*p* for interactions with diagnosis >0.05). The relative risk of sick leave associated with workplace bullying was 1.86 (95% CI: 1.30–2.82). This estimate was robust to adjustment for age, gender, education, chronic disease status, and contract type (temporary/long-term contract). Among hypothesized confounders, only days of hospitalizations were associated with productivity losses (Table 3). The adjusted marginal overall productivity cost of workplace bullying ranged from 13.9% (IBD) to 17.4% (PSO), corresponding to PPP 2010 US$ 4182–5236 yearly.

### 3.4. Workplace Bullying and Health-Related Quality of Life

**Figure 2:**
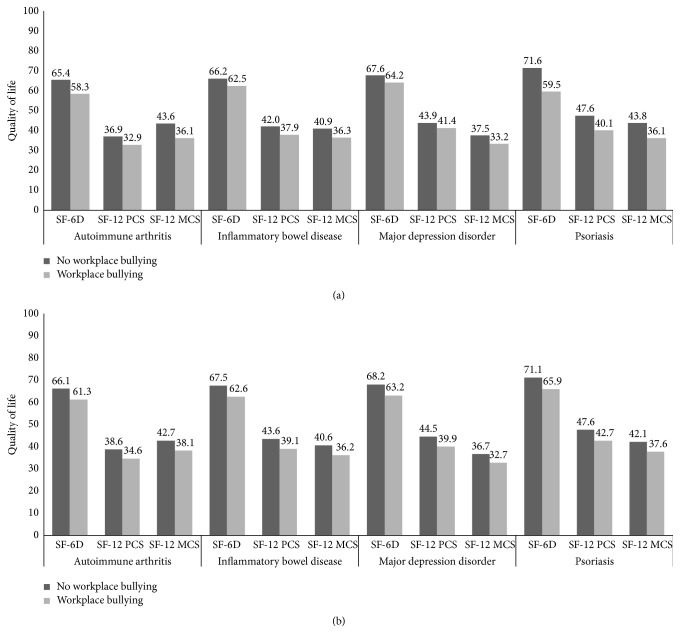
Health-related quality of life across workplace bullying status among workers with different chronic conditions. (a) Unadjusted scores. (b) Adjusted scores. Models included age, gender, time since diagnosis, time since workplace bullying onset, education, marital status, job demand, contract, diagnosis, and hospitalization days.

**Figure 3:**
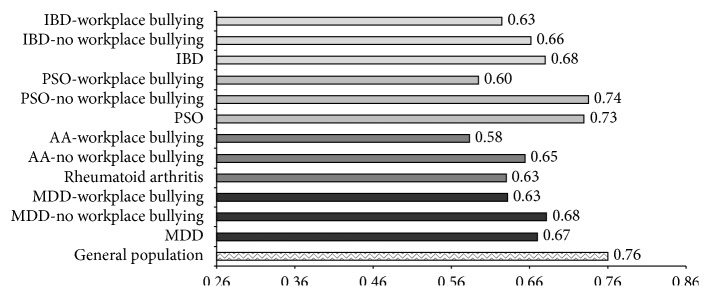
SF-6D scores in workers self-labelled as victims compared to those not reporting workplace bullying across different chronic diseases. Results from previous studies are reported for comparison [65–69].

The average scores of quality of life were 41.9 ± 10.6, 39.8 ± 11.3, and 0.664 ± 0.102 for the SF-12 PCS, SF-12 MCS, and SF-6D indexes, respectively. Among employed patients, 56.7% and 57.0% reported significant impairment as defined by the SF-12 PCS and SF-12 MCS scales, respectively. Workers who self-reported bullying at work were more likely classified as significantly impaired on both scales compared to nonvictims (SF-12 PCS: 55.5% versus 67.9%, *p* < 0.01; SF-12 MCS: 59.4% versus 74.3%, *p* < 0.01). Unadjusted health-related quality-of-life scores were associated with workplace bullying (Figure 2(a), *p* < 0.001). These associations were both robust to adjustment for possible confounders (Figure 2(b), *p* < 0.001) and were not moderated by disease status (*p* for interactions with diagnosis >0.05). The adjusted marginal disutility associated with workplace bullying ranged from 0.048 (AA) to 0.052 (PSO).  Figure 3 illustrates unadjusted SF-6D scores of workers who self-reported workplace bullying as compared to those who had not experienced workplace bullying.

Additionally, health-related quality-of-life scores were associated with days of hospitalization, gender, marital status, education, job security, and diagnosis (Table 3).

## 4. Discussion

### 4.1. Prevalence of Workplace Bullying

In this large multicenter cross-sectional study among workers with common chronic conditions [40–43], the prevalence of workplace bullying was 16% and most workplace bullying started after the onset of chronic disease. There is wide variation in prevalence estimates of workplace bullying across studies. Italian rates in the general working population range from 4.8% in a public service organization to 31.4% among airport employees [44]. Other prevalence studies have found rates of 3.5% in Sweden up to 27% in North America [45, 46]. These discrepancies are partially explained by different methods of measurement and criteria used to define workplace bullying [30, 47, 48].

### 4.2. Workplace Bullying and Productivity Loss

We observed a significant association between workplace bullying and all components of productivity. Workers who were not self-labelled as victims of workplace bullying showed WPAI scores similar to previous findings among patients with the same medical conditions [49–52]. However, participants who self-reported workplace bullying showed much higher WPAI scores. Our estimates suggest that the potential economic impact of preventive or therapeutic interventions addressing workplace bullying on yearly overall productivity loss might range from about PPP 2010 US$ 4200 to 5200 for each case prevented. Although cost-of-illness studies provide valuable information on the overall burden of disease, they generally lead to unrealistic expectations about savings from therapy as current treatments may reduce symptoms but are unable to eradicate the disease. Conversely, several effective interventions can be implemented at different levels to prevent and manage workplace bullying (e.g., antibullying policy, code of conduct, psychosocial risk analysis, and training) [53]. Coupled with the huge impact on overall productivity loss, workplace bullying should be considered an overriding issue for public health authorities and employers alike. Although our study cannot demonstrate causality of association, our findings help compare competing hypothetical scenarios to prioritize research investments. Our results demonstrate a strong association between sick-leave rates and exposure to workplace bullying. Nonetheless, previous studies have found relatively weak relationships between workplace bullying and absenteeism [15, 16]. This might be explained by the observation that victims of bullying may enhance their effort and commitment when their work performance and self-esteem are impaired [54]. Such compensative mechanism might not offset detrimental effects of bullying among victims with concurrent chronic health conditions due to the greater severity of their psychological and psychosomatic complaints [12].

Additionally, contrary to the short reference time adopted in this study (e.g., self-reported hours lost in the past week), most of the previous studies based their estimates on sick-leave events registered in administrative databases or adopted coarse self-reported measures (i.e., ever taken any sick leave due to workplace bullying) which may lead to information bias [26, 55, 56]. For example, as data repositories serve work-compensation procedures, their capture rate may be limited to events whose duration is eligible for compensation. For this reason, estimates from previous studies may underestimate the real productivity burden of workplace bullying. Consistent with labor supply models, there is empirical evidence suggesting that long-term sick leave is not an expression of withdrawal behaviors such as lateness, shorter sick leave, or reduced performance at work; on the contrary, longer spells are more frequently associated with serious illness rather than reduced commitment and motivation [57, 58]. Consistent with previous studies demonstrating the relationship between incivility at work and withdrawal behavior [59], we showed that workplace bullying is associated with reduced attendance (i.e., either lateness or sick days) beyond the effect of concurrent disabling medical conditions.

### 4.3. Workplace Bullying and Health-Related Quality of Life

A further important finding of our study was that workplace bullying was associated with worse health-related quality-of-life scores above and beyond the detrimental effect of other concurrent medical conditions. There is sparse evidence from previous studies that exposure to occupational psychosocial strain is associated with reduced health-related quality of life [60–63]. To our knowledge, this is the first study assessing the association between workplace bullying and health-related quality of life. The workplace bullying penalty observed in our study was clinically significant for the SF-12 PCS, SF-12 MCS, and SF-6D index according to the proposed thresholds for the minimal clinically important difference for HRQOL [64]. Additionally, the overall effect size observed in our study was similar to the SF-12 physical composite (Cohen's *d* = 0.42) compared to the SF-12 mental composite (Cohen's *d* = 0.47). Of note, victims of workplace bullying were more likely classified as significantly impaired on both SF-12 scales. The cut-off chosen represents the lowest octile of the score distribution in working populations and indicates a severely compromised function.

The overall effect size observed for the SF-6D index was moderate (*d* = 0.57): the adjusted disutility associated with workplace bullying corresponded to 18-19 days of healthy life lost for each year spent with the condition. The SF-6D scores reported by patients who were not self-labelled as victims of workplace bullying were comparable to figures reported in previous studies among subjects with the same medical condition (Figure 3) [65–69]. Exposure to workplace bullying is associated with posttraumatic stress reactions, anxiety, depression, and insomnia as well as chronic fatigue, psychosomatic symptoms, musculoskeletal and gastrointestinal disorders, headaches, and hypertension [8, 9, 12–14].

### 4.4. Strengths and Limitation

This study has several strengths. First, we evaluated the burden of workplace bullying on important outcomes among underresearched groups with different diseases. Second, we complied with recommendations for reporting economic evaluations in occupational medicine [29]. For example, in order to improve comparability and interpretability of our findings and to minimize the likelihood of underestimation, we adopted a widely used questionnaire, and we identified the source of price weights used and reported percent productivity loss for all components of indirect costs from the employer perspective [70]. By converting such findings into a financial metric, we sought to help organizational and public health stakeholders to better translate the impact of workplace bullying for people with chronic medical conditions. Third, our large sample size permitted adjustment for potentially important confounders thus reducing the likelihood of bias. Finally, community-based data on work productivity from a clinical population may present lesser degree of desirability bias compared to surveys conducted in occupational settings.

However, we must acknowledge some limitations. We relied on a self-labelling measure of workplace bullying, the most commonly adopted in epidemiological studies [48], which might have introduced information bias. How different estimation methods and measurements affect findings is still underinvestigated [30]. We primed participants with a widely accepted theoretic definition of workplace bullying to improve the accuracy of their subjective evaluation of victimization and power imbalance given the complexity of the phenomenon and potential for misinterpretation. Typically prevalence estimates yielded with the self-labelling approach are lower than those based on behavioral experience methods [47], so we used a broad cut-off for frequency of bullying experience (“now and then” to “many times a week”). Additionally, cross-sectional studies cannot prove causality since a necessary criterion of causation is the appropriate temporal relationship between the hypothesized risk factors and outcomes. Finally, we do not have information concerning the attrition rate of both studies. As a consequence, we cannot exclude the notion that selection bias may have occurred. However, the consistency of productivity loss and quality-of-life estimates found in our study with those published in the literature [49–52, 65–69] supports the validity of our results.

Future studies could collect data to assess psychosocial risk factors which may influence the associations observed. Although we did not observe any interaction between disease status and workplace bullying (i.e., the burden of bullying is consistent across different disease populations), our results may not be generalized to all workers with chronic conditions. Further studies could evaluate the humanistic and indirect burden of victimization at work among patients with an expanded range of chronic medical conditions (e.g., cardiovascular disease, diabetes, and chronic obstructive pulmonary disease).

## 5. Conclusions

Our findings demonstrate that the burden of workplace bullying on quality of life and productivity is substantial among workers with common and severe chronic diseases. These associations were independent of the underlying medical conditions (psoriasis, autoimmune arthritis, inflammatory bowel syndrome, and depression). This study provides key data to inform policy-making and prioritization of occupational health interventions.

## Figures and Tables

**Table 1:** Characteristics of study sample across different chronic diseases. *p* values represent significant levels of *χ* ^2^ for categorical variables, one-way ANOVA for continuous variables.

	Total (*N* = 1717)	AA (*N* = 295)	IBD (*N* = 449)	MDD (*N* = 700)	PSO (*N* = 273)	*p*
	*n* (%)	*n* (%)	*n* (%)	*n* (%)	*n* (%)	
Women (*N* = 1586)^*∗*^	978 (61.7)	172 (72.9)	243 (57.5)	436 (65.2)	127 (49.2)	<0.0001
Family (*N* = 1592)^*∗*^	969 (60.9)	170 (72.0)	240 (56.7)	376 (55.7)	183 (71.0)	<0.0001
University education (*N* = 1589)^*∗*^	315 (19.8)	45 (19.1)	78 (18.4)	116 (17.3)	76 (29.5)	0.0004
Employment (*N* = 1603)^*∗*^						<0.0001
Employed	897 (56.0)	129 (54.7)	268 (63.4)	334 (48.7)	166 (64.3)	
Inactive	293 (18.3)	20 (8.5)	62 (14.7)	177 (25.8)	34 (13.2)	
Retired	183 (11.4)	67 (28.4)	43 (10.2)	60 (8.75)	13 (5.04)	
Unemployed	230 (14.3)	20 (8.47)	50 (11.8)	115 (16.8)	45 (17.4)	
Contract (*N* = 1591)^*∗*^						<0.0001
Temporary	128 (8.05)	12 (5.17)	35 (8.29)	62 (9.13)	19 (7.36)	
Permanent	606 (38.1)	90 (38.8)	178 (42.2)	219 (32.3)	119 (46.1)	
Self-employed/employer	151 (9.49)	23 (9.91)	54 (12.8)	46 (6.77)	28 (10.8)	
Job demand (WAI) (*N* = 882)^*∗*^						<0.0001
Physical demand	80 (9.07)	4 (3.17)	17 (6.37)	47 (14.6)	12 (7.23)	
Mental demand	416 (47.2)	56 (44.4)	121 (45.3)	139 (43.0)	100 (60.2)	
Mixed demand	386 (43.8)	66 (52.4)	129 (48.3)	137 (42.4)	54 (32.5)	

	Mean (sd)	Mean (sd)	Mean (sd)	Mean (sd)	Mean (sd)	*p*

Age (*N* = 1603)^*∗*^	46.8 (13.1)	48.8 (10.4)	42.0 (12.2)	46.1 (10.9)	44.1 (8.79)	<0.0001
Workforce (*N* = 1717)^*∗*^	1603 (93.4)	236 (80.0)	423 (94.2)	686 (98.0)	258 (94.5)	<0.0001
Time since diagnosis (years) (*N* = 1593)^*∗*^	10.4 (10.2)	13.3 (10.1)	12.4 (9.45)	5.89 (7.38)	12.5 (11.5)	<0.0001
Hospitalization (days) (*N* = 1593)^*∗*^	2.37 (8.38)	1.90 (7.00)	3.50 (10.9)	2.55 (8.51)	0.72 (2.79)	0.0004

^*∗*^Number of valued cases for each variable.

**Table 2:** Sample characteristics across workplace bullying status. *p* values represent significant levels of *χ* ^2^ for categorical variables, one-way ANOVA for continuous variables.

	No workplace bullying *N* = 632 (83.7%) *n* (%)	Workplace bullying *N* = 123 (16.3%) *n* (%)	*p*
Women	367 (58.6)	68 (56.7)	ns
Married or *de facto*	392 (62.2)	67 (54.9)	ns
University education	154 (24.5)	22 (18.0)	ns
Job security	520 (84.1)	98 (15.9)	ns
Job demand			ns
Physical dem.	59 (86.8)	9 (13.2)	
Mental dem.	303 (85.1)	53 (14.9)	
Mixed dem.	261 (81.6)	59 (18.4)	

	Mean (sd)	Mean (sd)	*p*

Age	44.6 (10.9)	47.2 (12.3)	0.02
Time since diagnosis (years)	9.89 (9.71)	10.0 (9.97)	ns
Hospitalization (days)	1.61 (5.83)	1.88 (4.46)	ns

**Table 3:** Significant association estimates (*α* < 5%) between workers sociodemographic and clinical characteristics and study outcomes. Estimates represent the change score in outcome for each unit change in the independent variables. Models included age, gender, time since diagnosis, time since workplace bullying onset, education, marital status, job demand, contract, diagnosis, and hospitalization days.

	Impairment	Sick leave	Total productivity loss	SF-12 PCS	SF-12 MCS	SF-6D index
Hospitalization (days)	0.027^*∗*^	0.055^*∗∗∗*^	0.025^*∗*^	−0.012^*∗∗∗*^	−0.007^*∗∗*^	−0.005^*∗∗∗*^
Men	ns	ns	ns	0.069^*∗∗∗*^	ns	0.040^*∗∗∗*^
Living alone	ns	ns	ns	ns	−0.060^*∗∗*^	ns
High school or lower	ns	ns	ns	ns	ns	−0.028^*∗*^
Temporary work	ns	ns	ns	ns	−0.057^*∗*^	ns
Diagnosis						
AA	ns	ns	ns	−0.208^*∗∗∗*^	ns	−0.073^*∗∗∗*^
IBD	ns	ns	ns	−0.088^*∗∗∗*^	ns	−0.052^*∗∗*^
MDD	ns	ns	ns	−0.066^*∗∗*^	−0.139^*∗∗∗*^	−0.043^*∗∗*^
PSO	—	—	—	—	—	—

^*∗*^ *p* < 0.05; ^*∗∗*^ *p* < 0.01; ^*∗∗∗*^ *p* < 0.001.
